# Petermann’s Dental Almanach for 1879

**Published:** 1879-05

**Authors:** 


### Miscellaneous.

### PETERMANN'S DENTAL ALMANACH FOR 1879.

This elegant little volume of one hundred and twenty-eight pages, prepared with the author's accustomed accuracy, has again made its appearance—the third issue—and can be obtained at S. S. White's or other dental depots.

The almanach gives a complete list of all dental practitioners located in the German and Austria-Hungarian Empires. It is alphabetically arranged, and gives the full name, status and address of each individual.

Next, an alphabetical list of the cities, towns and places in which dentists are located; together with the number of inhabitants.

We also find an interesting statistical summary attached; according to which, there are four hundred and seventy-nine dentists (among them eight females) practising in Germany; among a population of forty-two million, seven hundred and fifty-seven thousand, eight hundred and twelve, located in one hundred and fifty-one cities and towns.

In Austria and Hungary, with a population of thirty-nine million, nine hundred and four thousand, four hundred and thirty-five, there are one hundred and thirty-three dentists, located in twenty-five cities.

Among these six hundred and twelve dentists of both empires, we find fifty-nine doctors of dental surgery (graduates of American dental colleges) and two "D. M. D.," (Harvard).

It contains also a complete catalogue of all dental journals monthly or quarterly, published in Germany, this and other countries.

The usual annual announcements of American dental colleges also find a place. In short, the little book is "brimful" of useful and interesting information.

As it is in German text, few American practitioners will be able fully to realize the value of the work. Yet the profession is largely indebted to Dr. Petermann for his efforts to shield the American dental colleges from the frauds which had been extensively practiced upon them by one John Buchanan, who—as "dean" of several "wild cat" institutions in this country—furnished bogus diplomas to any who might wish to purchase the same.

In attacking this great fraud Dr. Petermann has, of course made enemies among those who hold such diplomas, this is a natural consequence—but he certainly succeeded in severely checking the aforesaid barter of John Buchanan, "M. D." and Company.

It required no small amount of energy and disinterestedness, alone, and at his own pecuniary risk, to undertake this work. The American dental colleges especially, and, in fact, the whole profession of this country owe him their acknowledgments.

Dr. Petermann spent some time in this country, attended the requisite courses at the Philadelphia Dental College, and graduated therefrom in 1869. R.

